# A Platinum Resistance-Related lncRNA Signature for Risk Classification and Prognosis Prediction in Patients with Serous Ovarian Cancer

**DOI:** 10.1155/2022/7625138

**Published:** 2022-11-09

**Authors:** Yan Zhu, Jiongyu Chen, Li Zhou, Lina Zhang, Yuxin Liu, Yixuan Zhuang, Lin Peng, Yi-Teng Huang

**Affiliations:** ^1^Health Care Center, The First Affiliated Hospital of Shantou University Medical College, Shantou 515041, Guangdong, China; ^2^Medical Laboratory, Shenzhen Luohu People's Hospital, Shenzhen 518001, Guangdong, China; ^3^Central Laboratory, Cancer Hospital of Shantou University Medical College, Shantou 515041, Guangdong, China; ^4^Department of Gynecologic Oncology, Cancer Hospital of Shantou University Medical College, Shantou 515041, Guangdong, China; ^5^Department of Pathology, Cancer Hospital of Shantou University Medical College, Shantou 515041, Guangdong, China

## Abstract

Accurate risk stratification for patients with serous ovarian cancer (SOC) is pivotal for treatment decisions. In this study, we identified a lncRNA-based signature for predicting platinum resistance and prognosis stratification for SOC patients. We analyzed the RNA-sequencing data and the relevant clinical information of 295 SOC samples obtained from The Cancer Genome Atlas (TCGA) database and 180 normal ovarian tissues from the Genotype-Tissue Expression (GTEx) database. A total of 284 differentially expressed lncRNAs were screened out between platinum-sensitive and platinum-resistant groups by univariate Cox regression analysis. Then, a signature consisting of eight prognostic lncRNAs was used to construct a lncRNA score model by least absolute shrinkage and selection operator (LASSO) regression and multivariate Cox regression analysis. The ROC analysis showed that this signature had a good predictive performance for chemotherapy response in the training set (AUC = 0.8524) and the testing and whole sets with 0.8142 and 0.8393 of AUC, respectively. Dichotomized by the risk score of lncRNAs (lncScore), the high-risk patients showed significantly shorter progression-free survival (PFS) and overall survival (OS). Based on the final Cox model, a nomogram comprising the 8-lncRNA signature and 3 clinicopathological risk factors was then established for clinical application to predict the 1, 2, and 3-year PFS of SOC patients. The gene set enrichment analysis (GSEA) revealed that genes in the high-risk group were active in ATP synthesis, coupled electron transport, and mitochondrial respiratory chain complex assembly. Overall, our findings demonstrated the potential clinical significance of the 8-lncRNA-based classifier as a novel biomarker for outcome prediction and therapy decisions in SOC patients with platinum treatment.

## 1. Introduction

Ovarian cancer is the most lethal gynecological malignancy with a 5-year survival rate of 46% in women around the world [1]. According to the global cancer statistics 2020, there were an estimated 313,959 new cases and 207,252 cancer deaths worldwide in 2020, representing the 8th most commonly occurring cancer and the leading cause of cancer-related death among women worldwide. As the most prevalent histologic subtype, SOC accounts for over 70% of all ovarian cancers and is composed of high-grade and low-grade SOC. As for the treatment of ovarian cancer, platinum-based chemotherapy has been introduced to clinical practice as the backbone drug for more than 4 decades. Platinum, that directly interacts with DNA of cancer cells after hydration, has been employed as a therapeutic standard-of-care for ovarian cancer since the late 1970s [2]. Although 70% of ovarian cancer patients initially respond favorably to the first application of platinum-based chemotherapy, over 80% of them eventually relapse at some stage [3]. Chemoresistance remains the major obstacle to improving prognosis [4]. The outcome of patients with platinum resistance is generally poor, with a low response to further chemotherapy and a median survival of less than 12 months. Shortage of ideal platinum resistance-related biomarkers for risk classification and prognosis prediction is one of the major challenges in clinics.

Long noncoding RNA (lncRNA) is a class of RNA longer than 200 nucleotides that do not encode proteins and have no typical open reading frame [5]. It is now clear that lncRNAs participate in pathological processes by acting as enhancer RNAs (eRNAs), recruiting chromatin-modifying complexes, regulating the activity of transcription factors, and affecting the spatial conformation of chromosomes [6]. Accumulating evidence has highlighted that dysregulation of lncRNAs was involved in the chemoresistance and progression of ovarian cancer [7–9], and lncRNAs could represent novel biomarkers for prognostic signature in ovarian cancer [10, 11]. However, studies are limited regarding the association of lncRNAs signature and platinum chemoresistance in SOC. From this perspective, there should be some lncRNAs that could serve as risk stratification biomarkers for platinum resistance in ovarian cancer. In this study, we analyzed the sequencing datasets of SOC from TCGA and GTEx databases and identified a prognostic risk model consisting of eight platinum sensitivity-related lncRNAs. We also explored the potential molecular mechanisms and critical pathways that were associated with drug resistance in SOC induced by dysregulated lncRNAs.

## 2. Materials and Methods

### 2.1. Acquisition of Gene Expression and Clinical Data

The lncRNA sequencing profiles of 180 normal ovarian tissues from the GTEx database (https://www.gtexportal.org/home/datasets) and the normalized lncRNA sequencing and mRNA sequencing of 295 SOC samples from the TCGA dataset (https://portal.gdc.cancer.gov/repository?facetTab=cases) were downloaded. The normalized gene expression data of the TCGA-SOC database after log_2_-transformed were used for analysis. Samples from the TCGA database were divided randomly into a training set (*n* = 179) and an internal validation set (*n* = 116) at a ratio of 3 : 2 by the “caret” package Version 6.0–86 in *R* software (Version 4.0.2). The corresponding clinical data such as gender, age, pathologic grade, tumor-node-metastasis (TNM) stage, treatment type, treatment or therapy, prior malignancy, and survival data were also obtained from the TCGA.

Our inclusion criteria for the study cases were as follows: the primary tumor located in the ovary; histopathologically diagnosed with SOC; data for primary outcome evaluation and complete follow-up were available; the histological grade and clinical stage were clear; and all patients were treated with cisplatin. According to version 2.2020, National Comprehensive Cancer Network (NCCN) guidelines in ovarian cancer [12], 295 SOC cases extracted from the TCGA were divided into platinum-sensitive groups (nonrelapse and platinum-sensitive) and platinum-resistant groups (platinum-resistant and platinum-refractory). Nonrelapse means that no recurrence occurred in patients from the primary treatment until the end of follow-up. Patients who relapsed 6 months or more after initial chemotherapy were termed platinum-sensitive [13]. Those whose disease recurred in less than 6 months were termed platinum-resistant, whereas patients who were defined as platinum-refractory for cancer progression after two consecutive chemotherapy regimens without ever sustaining a clinical benefit were also classified in the platinum-resistant group [14]. The clinical benefit rate (CBR) is defined as without recurrence of ovarian cancer within 6 months in patients with complete remission (CR), partial remission (PR), or stable disease (SD) after primary therapy.

### 2.2. Analysis of Differentially Expressed lncRNAs

We integrated the expression matrices from the TCGA and GETx portal and identified the differentially expressed lncRNAs (DE-lncRNA) between SOC and normal tissues (marked as “TNlncRNAs”) using the *R* package “DESeq2” package (version 1.28.1) [15] and the DE-lncRNAs between the platinum-sensitive and platinum-resistant groups (marked as “RSlncRNAs”) using the *R* package “edgeR” (version 3.30.3) [16]. The *R* package “ggplot2” [17] (version 4.0.2) was then used to display the candidate DE-lncRNAs. The threshold for DE-lncRNA selection was based on a *P* value < 0.05 and |log_2_ (FC)| > 1. Then, we extracted the platinum-related lncRNAs based on the intersection set of TNlncRNAs and RSlncRNAs for further analysis using the “Venn” package (version 1.9).

### 2.3. Establishment and Validation of the Prognostic Gene Signature

To identify a multi-lncRNA signature with a good predictive performance for prognosis, DE-lncRNAs that were correlated with patients' PFS in the training set were screened using univariate Cox regression analysis. The lncRNAs with a Wald *P* value < 0.01 or |HR − 1| ≥ 0.35 were retained by the “survival” *R* package (version 4.0.2). The prognostic lncRNAs with nonzero coefficients were further screened by combining least absolute shrinkage and selection operator (LASSO) regression analysis with 10-foldcross-validation in the training set using the “glmnet” *R* package (Version 4.0.2).

### 2.4. Multi-lncRNA Signature Model Establishment and Validation

The risk coefficients (coeffs) for the candidate lncRNAs were subsequently calculated using a multivariate Cox proportional hazards regression analysis. We calculated the risk score of lncRNAs (lncScore) by the following formula:

(1) $$\begin{array}{c} lncScore=\sum {\mathrm{Exp}}_{lncRNAi}*{coef}_{lncRNAi}\text{,} \end{array}$$

where Exp_lncRNAi_ indicates the expression level of lncRNA in patients with SOC.

Then, the receiver operating characteristic (ROC) curve was drawn to assess the sensitivity and specificity of the hub lncRNAs for chemotherapy response in the training set by calculating the area under the curve (AUC) with the “heatmap” package [15]. The cutoff value of the risk score for classifying patients into the high-risk group and low-risk group was determined when the ROC curve reached the optimum sensitivity and specificity among the training cohort. Then, we validated its classification properties for chemotherapy response in the testing cohort and the whole cohort. Kaplan–Meier curve was performed to evaluate the survival time of the high‐risk and low‐risk groups, and then, the log-rank method was used to verify the results.

### 2.5. Independent Prognosis Analysis and Nomogram Construction

To verify the efficacy of the candidate lncRNA signature in predicting the prognosis and clinicopathological features such as age at diagnosis, clinical stage, pathological grade, tumor residual disease, and venous invasion, univariate and multivariable Cox regression analyses were used to select independent prognostic factors. The cox regression coefficients were introduced to generate nomograms with the “rms” package version 6.0–0 [18] in *R* software (version 4.0.2). The survival probability estimated by Kaplan–Meier analysis was used to correct the nomogram [19].

### 2.6. Gene Set Enrichment Analysis (GSEA)

The 295 SOC samples were divided into the high-risk and low-risk groups according to the lncScore threshold. The “edgeR” package was used to analyze the DE-mRNA expression profile between the two groups with FDR <0.05 and |log_2_FC| > 1. The “clusterProfiler” package (version 3.16.1) in *R* software (version 4.0.2) was employed to perform GSEA [20] for the significantly altered gene ontology (GO) function. *P* < 0.01 was considered as the cutoff criterion. Finally, the “enrichplot” package (version 1.8.1) in *R* software (version 4.0.2) was used to demonstrate the enrichment functions that were statistically significant.

## 3. Results

### 3.1. The Study Flowchart and Patient Clinicopathological Features

**Figure 1:**
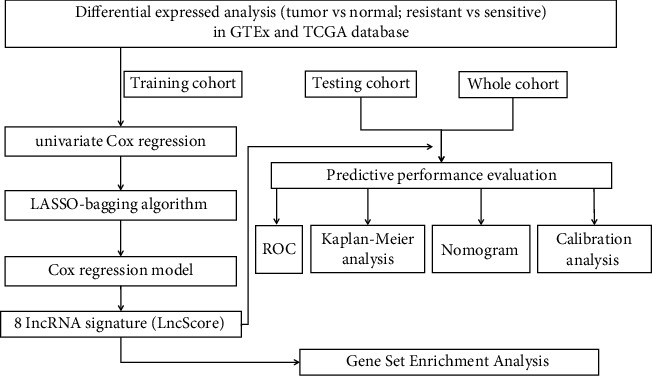
Study flowchart of the analytical procedure. GTEx, the genotype-tissue expression; TCGA, The Cancer Genome Atlas; LASSO, least absolute shrinkage and selection operator; ROC, receiver operating characteristic.

A detailed data processing flowchart is shown in Figure 1. The 304 SOC samples with primary therapy outcome were retained. These samples consisted of 212 cases in the platinum-sensitive group, which included 52 cases of nonrecurrence and 160 cases of platinum-sensitive, with a median age of 58 years old (IQR, interquartile range: 51–67) and 83 cases in the platinum-resistant group which included 35 cases of platinum-resistant and 48 cases of refractory, with a median age of 59 years old (IQR: 50–67). The clinicopathological characteristics of the patients in the training, testing, and whole cohorts are summarized in Table 1.

### 3.2. Screening for lncRNAs Associated with Platinum-Based Response from the TCGA and GTEx Databases

**Figure 2:**
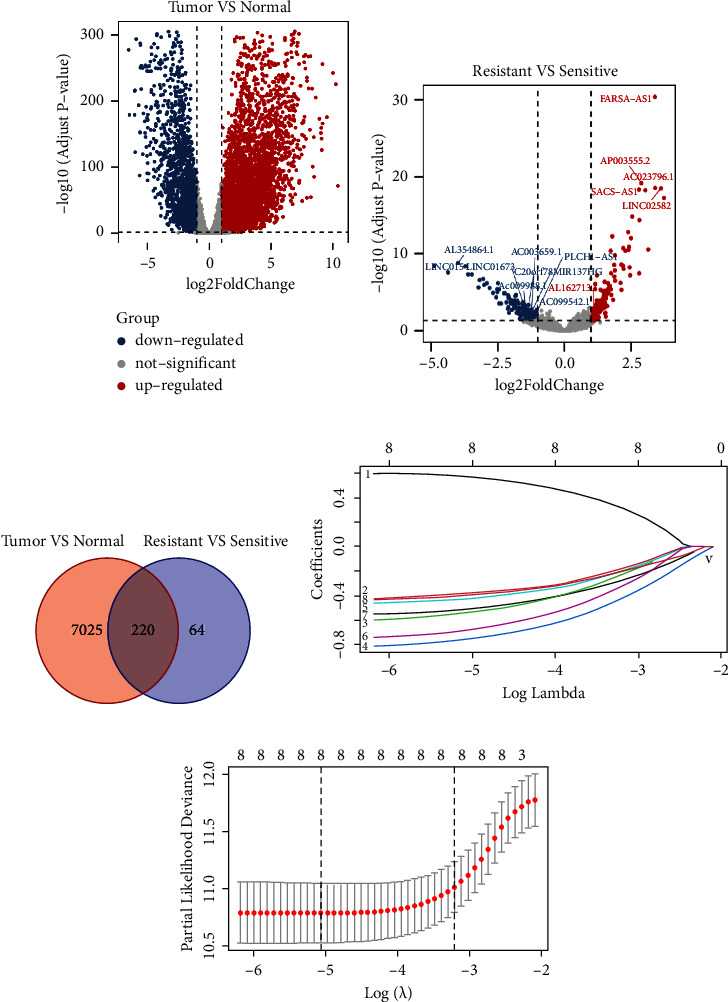
Selection of candidate lncRNAs from TCGA and GTEx databases. ((a), (b)) The volcano plot showing the distribution of differentially expressed lncRNAs between TNlncRNAs (a) and between RSlncRNAs (b) The red, blue, and grey points represent the upregulated, downregulated, and nondifferentially expressed lncRNAs, respectively. (c) A Venn diagram between TNlncRNAs and between RSlncRNAs. (d) LASSO regression coefficient profiles of the eight potential lncRNAs. (e) Plot of the cross-validation error rates.

Based on the bioinformatics analysis, a total of 7245 DE-lncRNAs (5247 upregulated lncRNAs and 1998 downregulated lncRNAs in tumor tissues) between normal and tumor tissues were identified, as shown in Figure 2 (Figure 2(a)). Similarly, a total of 284 DE-lncRNAs were identified between the sensitive and resistant groups (126 upregulated lncRNAs and 158 downregulated lncRNAs in the platinum-resistant group) (Figure 2(b)). The intersection set of TNlncRNAs and RSlncRNAs got a final 220 platinum-related lncRNAs (Figure 2(c), Supplemental Table S1).

By using a univariate Cox regression analysis, eight lncRNAs were found to be significantly associated with PFS (*P* value < 0.01 and |HR-1| > 0.35) and were defined as the prognostic lncRNAs in the training set (Table 2). The prognostic signature was composed of the following lncRNAs: LINC01673, MIR137HG, PLCH1-AS1, AC009988.1, C20orf78, AC003659.1, AL162713.1, and AC099542.1. Among them, AL162713.1 presented as a risk factor (HR > 1) for poor PFS, while the other seven lncRNAs were protective factors for cancer progression in patients with SOC (HR < 1) (Table 2).

### 3.3. Construction of the Eight lncRNAs-Based Prognostic Model

We finally identified eight markers with LASSO coefficient >0.1 and developed a risk score formula by a linear combination of the expression values of the eight lncRNAs weighted by multivariate Cox regression coefficients (Figures 2(d) and 2(e); Table 2). The risk score for each patient was calculated based on the following formula:

(2) $$\begin{array}{c} lncScore={\mathrm{Exp}}_{LINC01673}\times \left(-0.46\right)+{\mathrm{Exp}}_{MIR137HG}\times \left(-0.45\right)+{\mathrm{Exp}}_{PLCH1-AS1}\times \left(-0.63\right)+{\mathrm{Exp}}_{AC009988.1}\times \left(-0.84\right)+{\mathrm{Exp}}_{C20orf78}\times \left(-0.48\right)\\ +{\mathrm{Exp}}_{AC003659.1}\times \left(-0.77\right)+{\mathrm{Exp}}_{AL162713.1}\times 0.61+{\mathrm{Exp}}_{AC099542.1}\times \left(-0.57\right)\text{.} \end{array}$$

**Figure 3:**
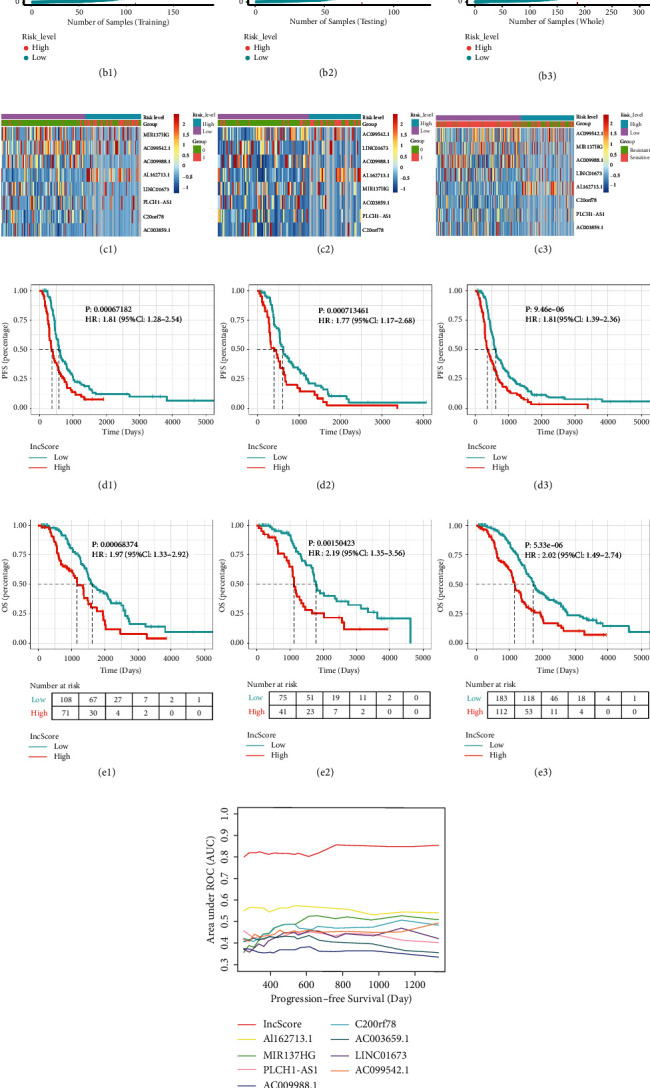
Construction and validation of the eight lncRNAs-based prognostic model. (a) ROC curve for the efficacy prediction of platinum-based chemotherapy in the training set (a1), testing cohort (a2), and the whole cohort (a3). (b) PFS status scatter plots and risk score distribution for patients in the training set (b1), testing cohort (b2), and the whole cohort (b3). Red and blue indicate high-risk and low-risk scores, respectively. (c) A heatmap for the 8-lncRNA signature in the training set (c1), testing cohort (c2), and the whole cohort (c3), and red and blue indicate high and low expression, respectively. Kaplan–Meier curves of PFS (d) and OS (e) for the high- and low-risk patients grouped by the 8-lncRNAs signature in the training set (d1, e1), testing cohort (d2, e2), and the whole cohort (d3, e3). The time-dependent AUC analysis of the individual lncRNA and lncScore for predicting PFS (f) and OS (g) in the whole set.

ROC curves were plotted to evaluate the predictive power based on specificity and sensitivity. The AUC of ROC for the prognostic signature is 0.8534, which shows excellent performance for the efficacy prediction of platinum-based chemotherapy. The cutoff value of the risk score for classifying patients into the high‐risk group and low‐risk group was determined when the ROC curve reached the optimum sensitivity (75.8%) and specificity (82.4%) in the training cohort (Figure 3). The distribution of the risk score was plotted along with the corresponding survival outcome (Figure 3(b1)). The expression of the eight lncRNAs in the training set is shown in Figure 3(c1). Kaplan–Meier analysis showed that patients in the high-risk group had worse PFS than those in the low-risk group in the training cohort (HR = 1.81; 95% CI: 1.28–2.54; *P* = 0.0006; Figure 3(d1)). Furthermore, similar results were obtained for the lncScore on OS (HR = 1.97; 95% CI: 1.33–2.92; *P* = 0.00068; Figure 3(e1)).

### 3.4. Validation of the Eight lncRNAs-Based Prognostic Model

To verify the predictive performance of this 8-lncRNA model, we divided patients from the testing and whole cohorts into high-risk and low-risk groups according to the cutoff value determined in the training cohort. The AUC of patients in the testing and the whole sets was 0.8023 and 0.8344, respectively (Figures 3(a2) and 3(a3)), implying that the lncScore of this model has a good performance in predicting the efficacy of platinum-based chemotherapy.

The distribution of risk scores and survival status of patients in the testing and whole cohorts are shown in Figures 3(b2) and 3(b3). The expression of these eight lncRNAs in the testing and whole cohorts is shown in Figures 3(c2) and 3(c3). In the testing cohort, patients with a high lncScore had a poor PFS (HR = 1.77; 95% CI: 1.17–2.68; *P* = 0.0071; Figure 3(d2)) and a short OS (HR = 2.19; 95% CI: 1.35–3.56; *P* = 0.0015; Figure 3(e2)). Likewise, a high lncScore was associated with short PFS (HR = 1.81; 95% CI: 1.39–2.36; *P* < 0.0001; Figure 3(d3)) and OS (HR = 2.02; 95% CI: 1.49–2.74; *P* < 0.0001; Figure 3(e3)) in the whole cohort. The time-dependent ROC analysis indicated the area under the ROC curve of the lncScore was the biggest for PFS and OS prediction compared with a single lncRNA alone in the whole set data (Figures 3(f) and 3(g)).

### 3.5. Establishment and Validation of a Nomogram

**Figure 4:**
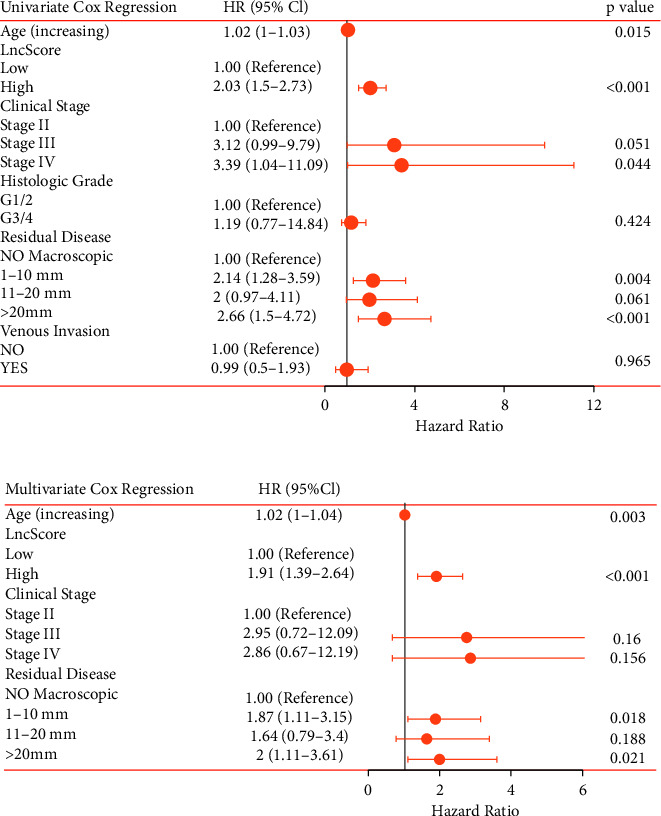
Forest plot for clinical characteristics and lncScore related to SOC prognosis in univariate and multivariate Cox analyses. Identification of possible risk factors for poor OS in SOC by the univariate Cox analysis. (a) Multivariate Cox analysis (b). The coordinate of the solid circle represents the hazard ratio, and the length of the line represents the 95% confidence interval. lncScore, the risk score of lncRNAs.

Univariate Cox regression analysis was performed to assess the possible risk factors for SOC patients, including age at first diagnosis, clinical stage, venous invasion, pathological grade, and tumor residual disease (Figure 4) (Figure 4(a)). The results showed that the clinical stage III/IV, increased risk score and age, and residual disease >20 mm were related to poor prognosis of SOC patients. Afterward, according to the multivariate Cox analysis, lncScore (HR = 1.91; 95% CI: 1.39–2.64; *P* < 0.001), age at diagnosis (HR = 1.02; 95% CI: 1.00–1.04; *P* = 0.003), and tumor residual disease with 1–10 mm (HR = 1.87; 95% CI: 1.11–3.15; *P* < 0.001) and >20 mm (HR = 2.00; 95% CI: 1.11–3.61; *P* = 0.021) were identified as independent prognostic factors for OS in the whole cohort (Figure 4(b)).

**Figure 5:**
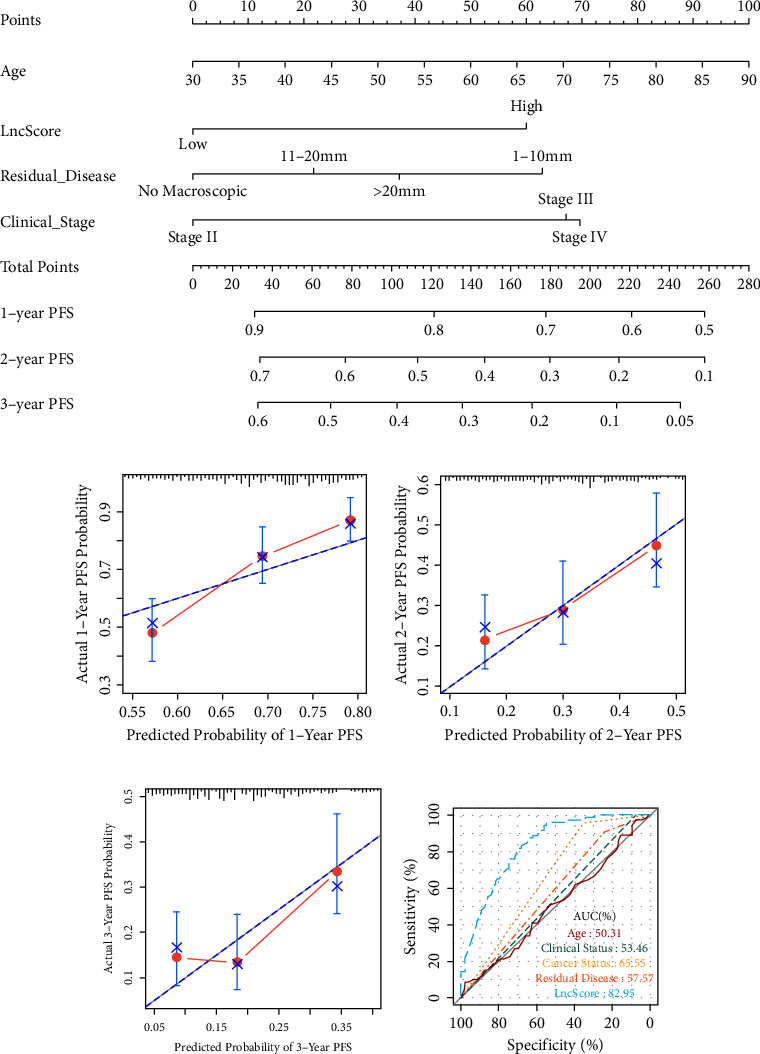
Construction and validation of the lncScore clinicopathologic nomogram for PFS prediction in SOC patients. (a) A nomogram for predicting 1, 2, and 3-year PFS of SOC patients. ((b)-(d)). Calibration curves of the nomogram for 1-year PFS (b), 2-year PFS (c), and 3-year PFS (d) in the whole cohort. (e) The ROC plot is platinum sensitivity prediction in the whole set. AUC, area under the curve.

To develop a clinically applicable tool that can provide individualized estimates of 1, 2 or 3-year PFS and 3 or 5-year OS, a nomogram was subsequently established based on the final Cox model for PFS and OS. As shown in Figure 5 (for PFS) and Supplementary Figure S1 (for OS), the lncScore takes the biggest weight for prediction, followed by age at diagnosis, clinical stage, and the tumor residual disease. Most important, the calibration plots of the nomogram predicting PFS and OS performed well with the ideal model (Figures 5(b)–5(d), Figures S1(b) and S1(c)).

Likewise, the lncScore showed the strongest predictive power in distinguishing platinum-sensitive and platinum-resistance patients with SOC (AUC = 0.8295), whereas the AUC-ROC for postoperative tumor residual disease, clinical stage, age at diagnosis, and pathological grade ranged from 0.5031 to 0.6555 (Figure 5(d)).

### 3.6. Functional Analysis of the Prognostic lncRNA Model

**Figure 6:**
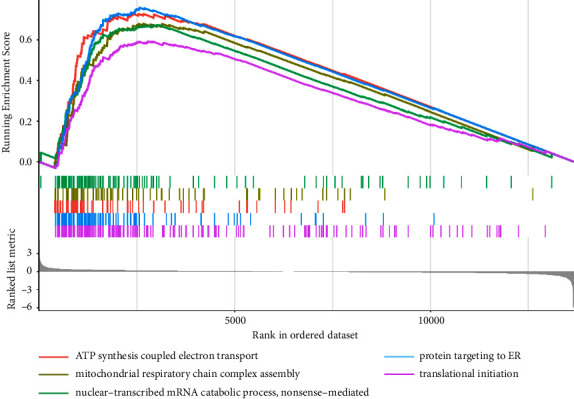
GSEA delineation of the biological pathways related to the risk score values of the lncRNA model. GSEA, gene set enrichment analysis. Colour images are available online.

GSEA was carried out to explore the biological effects of this lncRNA model, and our results suggested that the high score of lncRNAs in the model showed significant enrichment in a crowd of biological process terms, mainly involved in ATP synthesis coupled electron transport, mitochondrial respiratory chain complex assembly, nuclear-transcribed mRNA catabolic process (nonsense-mediated), protein targeting to ER, and translational initiation (Figure 6).

## 4. Discussion

The existing FIGO (International Federation of Gynecology and Obstetrics) and AJCC (American Joint Committee on Cancer) cancer staging systems, as well as pathological grade, are the major factors affecting the therapeutic regimen decision, but they could not give enough information in predicting platinum resistance for patients with SOC. In recent years, dysregulated lncRNAs have been documented to be involved in tumor progression and have a great potential in the diagnosis and prognosis of ovarian cancer as novel independent molecular biomarkers [21]. There is growing evidence that lncRNAs could serve as potential biomarkers for the prediction of platinum-based chemoresistance [22–24]. Several studies have shown the potential of a lncRNA expression signature for predicting the chemotherapeutic sensitivity of ovarian cancer while narrowly restraining its cohort in advanced stages or high-grade SOC [25, 26]. To date, the lncRNA expression profiles-based prognostic signature for the prediction of platinum sensitivity in SOC patients have not been developed.

In this study, a comprehensive analysis of lncRNA expression profiles was conducted to discriminate platinum-based chemotherapeutic outcomes in patients with SOC. Eight lncRNAs with predictive signatures of platinum-based chemotherapeutic sensitivity were identified in a large number of SOC patients from the TCGA. Among these lncRNAs, an increased expression level of AL162713.1 was associated with a poor PFS, and the other 7 lncRNAs were positively correlated with PFS time. We validated the predictive power of this 8-lncRNA signature on an internal validation set of 116 TCGA patients as well as on the entire TCGA cohort. The AUC value of the prognostic signature was 0.8295, which was similar to the previous lncRNA panel for platinum-resistance prediction in patients with high-grade SOC developed by Song et al. [26], whereas higher than those developed are presented in two published studies in advanced stage and high-grade SOC patients [21, 25]. In stratification of the subjects according to the cutoff value of lncScore, the survival curves showed a high level of consistency among the three datasets, which indicates that SOC patients with higher scores have a shorter duration of PFS and OS than patients in the lower score group. Moreover, the 8 core lncRNAs in this prognostic panel were found to be more reliable and accurate than a single lncRNA alone. This finding is consistent with the common perception that a multiple noncoding RNA panel is generally more precise and robust in predicting the outcomes of patients with cancer [27].

With further validation, the subsequent multivariate Cox regression analysis showed that the prognostic signature value of the lncScore functioned as an independent risk factor for poor prognosis in SOC patients besides age at diagnosis and tumor residual disease >20 mm. Based on the final Cox model, a nomogram was established for clinical use by integrating the 8-lncRNA signature and three clinicopathological risk factors for the clinical assessment of the 3- and 5-year PFS and OS probabilities for SOC patients. And from the nomogram, we can see that the patient with a higher lncScore, or with residual tissue of 1–10 mm or >20 mm, or higher clinical staging, would have a higher total of points and get a higher probability of disease progression in 1, 2, or 3 years postsurgery. Notably, the AUC-ROC value of the lncScore in the nomogram showed excellent prognostic value in predicting the efficacy of platinum-based chemotherapy compared with that of other existing risk factors, including postoperative tumor residual disease, clinical stage, and age at diagnosis, implying that this 8-lncRNA signature could be a clinically applicable tool for individualized estimation and guidance of clinical decision-making regarding the usage of platinum-based chemotherapy.

Among the eight lncRNAs with this signature, only MIR137HG has been illustrated in some literature. MIR137HG (gene ID: 400765) is the host gene for MIR137. It was demonstrated that recruiting DNA methyltransferase DNMT3a to the promoter of MIR137 in colorectal cancer cells could lead to suppression of MIR137 expression [28]. Earlier studies focused on its mutation involved in the development of liver cancer [29]. Recently, MIR137HG was introduced to be one of the lncRNA signatures for predicting the survival of patients with hepatocellular carcinoma [30]. Additionally, MIR137HG was identified as a core lncRNA included in the tumorigenesis-related ceRNA network and as a potential prognostic biomarker for laryngeal squamous cell carcinoma and muscle-invasive bladder cancer [31, 32]. It is with regret that existing research rarely reported the biological functions of MIR137HG in cancer. Lyu et al. performed GO and KEGG enrichment analyses and revealed that MIR137HG may function as an oncogene in muscle-invasive bladder cancer by regulating epithelial cell differentiation, cytokine production, the PPAR signaling pathway, and TNF signaling pathway [31]. In the study by Zhong et al., MIR137HG was identified as an immune-related lncRNA and used in a prognostic lncRNA signature in neuroblastoma, which was mainly enriched in cancer-related pathways and immune-related pathways [33]. These results are in line with what was found in this study, which suggests that MIR137HG might be an lncRNA that affects the prognosis in pan-cancers. 

Afterward, an in-depth study by GSEA enrichment analysis for the characteristics and biological behavior of the constructed lncRNA model was performed to understand the potential mechanisms involved in platinum resistance in SOC. Earlier evidence indicated that intracellular ATP-dependent processes contributed to cisplatin resistance of tumor cells, and the elevated ATP level appeared to be a consequence of enhanced mitochondrial ATP production [34]. Meanwhile, ATP-binding cassette transporters were implicated in the influx or efflux of platinum-based chemotherapeutic agents [35]. Consistent with the literature, the current research found that the high-risk group of chemoresistance was more active in ATP synthesis coupled electron transport, further supporting this signature's involvement in platinum resistance.

Mitochondria are known to play a central role in regulating cellular metabolism and producing adenosine triphosphate (ATP). Interestingly, mitochondrial respiratory chain complex assembly was also found to be active in the high-risk group. It was documented that mitochondrial defects and the dysfunctions of energy production contributed to the resistance to platinum drugs in cancers [36], ovarian cancer included [37]. This finding is consistent with that of Dong et al., who indicated that mitochondrial dysfunction was associated with platinum resistance [38].

In addition, GSEA results showed that some diverse functions, like NMD, were clustered in the high-risk group. NMD is a conserved mRNA surveillance mechanism that triggers the degradation of aberrant mRNAs harboring premature termination codons. In some cases, tumors exploited NMD by positively selecting for nonsense mutations to downregulate tumor suppressor genes, as has been shown for BRCA1 and BRCA2, which were associated with inherited susceptibility and platinum resistance in ovarian cancer [39–43]. However, the findings of the current study do not support these two following studies. Although BRCA1-Δ11q alternative splice isoform has been shown to contribute to cisplatin-resistance in ovarian cancer, the alternative splicing rate or BRCA1-Δ11q level was not altered by frameshift mutations in exon 11-induced NMD or its containing-transcripts. Another recent research demonstrated that inhibition of NMD induced by stress could lead to upregulation of the cystine/glutamate exchanger SLC7A11 [44], which has been associated with platinum resistance in ovarian cancer [45]. Accordingly, this result needs to be interpreted with caution.

The present results indicated that the lncScore model based on the eight lncRNAs can effectively stratify the patients with SOC into platinum-sensitive and -resistant groups, which updated the current prognostic risk prediction model for platinum resistance. Almost inevitably, there were some shortcomings in the current study. First, the cases were collected only from the TCGA database without other independent datasets, which might be skewed by selection bias. Furthermore, this study employed a retrospective design, so it requires prospective cohorts in clinical trials to further address this issue. And third, the biological function of the lncRNAs included in this 8-lncRNA signature has not been fully characterized before, and further functional studies, both in vitro and in vivo, are required.

In summary, by analyzing the available transcriptome sequencing data from the public data repositories, our study presents a panel of platinum sensitivity-related 8-lncRNA signatures with superior accuracy for prognosis and good predictive performance in SOC patients with platinum treatment. Furthermore, a nomogram combining the panel of these 8 lncRNAs with clinicopathologic characteristics showed excellent calibration consistency, improving the model's practicality for clinical applications. In view of the contribution to drug resistance-related biological processes, 8 lncRNAs could be potential therapeutic targets.

## Figures and Tables

**Table 1:** Clinical characteristics of patients with serous ovarian cancer involved in this study.

Characteristics	Training cohort	Testing cohort	Whole cohort
	*n* = 179	*n* = 116	*n* = 295
Age, median (IQR), *y*	58 (50–67)	58 (51–68)	58 (50–67)
Sex, *n* (%)			
Female	179 (100)	116 (100)	295 (100)
Clinical stage, *n* (%)			
Stage II	13 (7.26)	5 (4.31)	18 (6.10)
Stage III	139 (77.65)	94 (81.03)	233 (78.98)
Stage IV	27 (15.09)	17 (14.66)	44 (14.92)
Residual disease, *n* (%)			
No macroscopic	32 (17.88)	24 (20.69)	56 (18.98)
1–10 mm	88 (49.16)	53 (45.69)	141 (47.80)
11–20 mm	11 (6.15)	8 (6.90)	19 (6.44)
>20 mm	30 (16.76)	22 (18.96)	52 (17.63)
*X*	18 (10.05)	9 (7.76)	27 (9.15)
Cancer status, *n* (%)			
Tumor free	45 (25.14)	30 (25.86)	75 (25.42)
With tumor	126 (70.39)	81 (69.83)	207 (70.17)
*X*	8 (4.47)	5 (4.31)	13 (4.41)
Histologic grade, *n* (%)			
G1/2	20 (11.17)	19 (16.38)	39 (13.22)
G3/4	159 (88.83)	97 (83.62)	256 (86.78)
Venous invasion, *n* (%)			
No	25 (13.97)	13 (11.21)	38 (12.88)
Yes	45 (25.14)	33 (28.45)	78 (26.44)
*X*	109 (60.89)	70 (60.34)	179 (60.68)
Primary therapy outcome, *n* (%)			
Complete remission	129 (72.07)	77 (66.38)	206 (69.83)
Partial remission	24 (13.41)	17 (14.66)	41 (13.90)
Stable disease	11 (6.14)	10 (8.62)	21 (7.12)
Progressive disease	15 (8.38)	12 (10.34)	27 (9.15)

IQR1/4, interquartile range; *y*, year; *X*, unknown.

**Table 2:** The eight lncRNAs screened out by a univariate Cox regression and a signature established by a multivariate Cox regression (*n* = 295).

Gene ID	Gene symbol	Chromosome	Univariate Cox regression		Multivariable Cox regression		
			HR (95% CI)	*P* value	HR (95% CI)	Coefficient	*P* value
ENSG00000234052	LINC01673	chr21: 27638613–27675024(+)	0.53 (0.36–0.78)	0.001	0.63 (0.43–0.93)	−0.46	0.02
ENSG00000225206	MIR137HG	chr1: 97933474–98049863 (+)	0.61 (0.44–0.84)	0.002	0.64 (0.48–0.86)	−0.45	0.003
ENSG00000239508	PLCH1-AS1	chr3: 155449184–155457753 (+)	0.52 (0.33–0.82)	0.005	0.53 (0.33–0.85)	−0.63	0.009
ENSG00000223432	AC009988.1	chr10: 121615425–121615839 (−)	0.49 (0.33–0.71)	<0.001	0.43 (0.29–0.64)	−0.84	<0.001
ENSG00000149443	C20orf78	chr20: 18809728–18830153 (−)	0.45 (0.26–0.78)	0.004	0.62 (0.37–1.04)	−0.48	0.071
ENSG00000237994	AC003659.1	chrX: 28942050–28942568 (+)	0.44 (0.24–0.78)	0.006	0.46 (0.25–0.84)	−0.77	0.011
ENSG00000270522	AL162713.1	chr13: 43787591–43787852 (+)	1.36 (1.09–1.70)	0.006	1.84 (1.47–2.31)	0.61	<0.001
ENSG00000241593	AC099542.1	chr3:81246579–81297345 (−)	0.59 (0.44–0.79)	<0.001	0.57 (0.42–0.77)	−0.57	<0.001

## Data Availability

Publicly available datasets were analyzed in this study. The data used to support the findings of this study are available at https://portal.gdc.cancer.gov/.

## Supplementary Materials

Supplementary Materials Figure S1: Construction and validation of the lncScore-clinicopathologic nomogram for survival prediction in SOC patients. Table S1: List of the 220 platinum-related lncRNAs based on the intersection set of TNlncRNAs and RSlncRNAs.
