# Supplementary material for: A Platinum Resistance-Related lncRNA Signature for Risk Classification and Prognosis Prediction in Patients with Serous Ovarian Cancer
Source: J Oncol. 2022 Nov 9;2022:7625138. doi: 10.1155/2022/7625138 (PMC10202609; doi:10.1155/2022/7625138)

Fig.S1 Construction and validation of the lncScore-clinicopathologic nomogram for survival prediction in SOC patients.

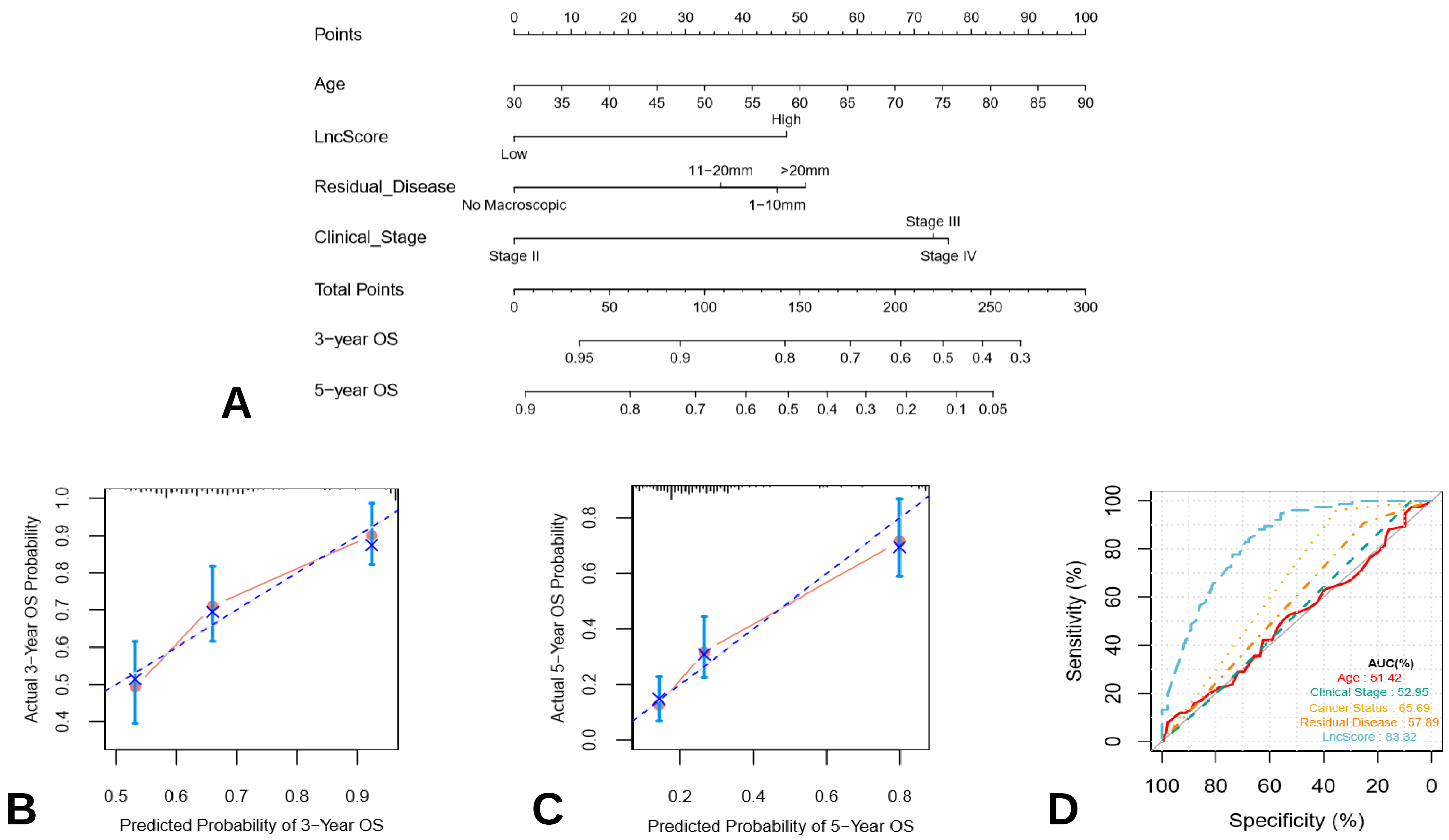

Supplement: Supplementary Materials — Figure S1: Construction and validation of the lncScore-clinicopathologic nomogram for survival prediction in SOC patients. Table S1: List of the 220 platinum-related lncRNAs based on the intersection set of TNlncRNAs and RSlncRNAs. [file 7625138.f1.zip › supplementary figure S1.pdf]
