# Supplementary material for: A Platinum Resistance-Related lncRNA Signature for Risk Classification and Prognosis Prediction in Patients with Serous Ovarian Cancer
Source: J Oncol. 2022 Nov 9;2022:7625138. doi: 10.1155/2022/7625138 (PMC10202609; doi:10.1155/2022/7625138)
Supplement: Supplementary Materials — Figure S1: Construction and validation of the lncScore-clinicopathologic nomogram for survival prediction in SOC patients. Table S1: List of the 220 platinum-related lncRNAs based on the intersection set of TNlncRNAs and RSlncRNAs. [file 7625138.f1.zip › TableS1 DE-lncRNA (1).pdf]

NO.	row	Tumor vs Normal						Sensitive vs Resistant				
		baseMean	L2FC	lfcSE	stat	PValue	padj	L2FC	logCPM	F	PValue	FDR
1	A2ML1-AS2	0.5233174	2.108088242	0.36779	5.731773	9.94E-09	1.69E-08	-1.890933194	2.285373	23.74049	1.78E-06	0.000195
2	AC002115.1	0.9482178	2.074669713	0.300358	6.90733	4.94E-12	9.29E-12	-1.211058699	2.214297	13.4462	0.00029	0.012719
3	AC002351.1	5.9810215	5.443695858	0.42683	12.75379	2.97E-37	9.29E-37	1.449296056	4.459626	13.4236	0.000295	0.012863
4	AC003659.1	0.2955067	1.41938787	0.479103	2.962595	0.003051	0.004158	-1.267116142	1.573704	19.15345	1.66E-05	0.001207
5	AC003975.1	0.2922065	1.45496472	0.439706	3.308948	0.000936	0.001314	1.485683903	1.736217	28.48652	1.86E-07	2.66E-05
6	AC004147.2	0.8899717	2.273168593	0.471919	4.816857	1.46E-06	2.31E-06	-1.574881907	2.132151	16.56404	6.01E-05	0.003503
7	AC004835.1	1.7002303	1.335300643	0.216247	6.174896	6.62E-10	1.17E-09	-1.838362196	2.946622	28.50111	1.84E-07	2.66E-05
8	AC004920.1	0.4998541	2.27632673	0.479423	4.748056	2.05E-06	3.23E-06	1.174780917	1.781475	17.4472	3.87E-05	0.002432
9	AC005307.1	17.708781	6.660967751	0.308663	21.58008	2.76E-103	2.03E-102	-1.366123713	5.722839	11.30175	0.000877	0.028773
10	AC005487.1	1.6024283	3.259393291	0.322554	10.10497	5.25E-24	1.31E-23	1.676043362	2.724355	31.02566	5.64E-08	9.53E-06
11	AC005614.1	2.432151	1.972074734	0.210632	9.362651	7.78E-21	1.81E-20	-1.061534316	2.699559	15.69055	9.31E-05	0.004972
12	AC006058.3	0.9743284	2.210640884	0.341151	6.479958	9.17E-11	1.66E-10	1.031189648	2.1657	11.95701	0.000623	0.022675
13	AC006065.4	0.6475368	2.310973191	0.450367	5.131316	2.88E-07	4.67E-07	-1.096529001	1.883929	10.06181	0.001669	0.047401
14	AC006262.3	0.9278177	3.056215914	0.522852	5.845278	5.06E-09	8.69E-09	1.329299538	2.125913	15.94052	8.21E-05	0.004551
15	AC006372.1	2.0792265	2.617007734	0.302561	8.649513	5.17E-18	1.14E-17	-1.20575859	2.767843	11.85789	0.000656	0.02326
16	AC006372.2	1.1103049	1.779674512	0.347488	5.121544	3.03E-07	4.91E-07	1.300543131	2.437959	15.3732	0.000109	0.005756
17	AC007099.1	0.8709594	2.758403129	0.246315	11.19869	4.14E-29	1.13E-28	-1.819370893	2.565099	28.44351	1.89E-07	2.67E-05
18	AC007422.1	5.4011611	5.324937411	0.302342	17.61231	1.98E-69	9.92E-69	-1.187001397	3.705597	11.88666	0.000647	0.023224
19	AC007423.1	0.5613144	2.025395072	0.400801	5.053369	4.34E-07	7.00E-07	2.133615688	2.100209	50.78222	7.61E-12	3.63E-09
20	AC007922.3	0.9719353	3.145594989	0.529687	5.938592	2.87E-09	4.98E-09	-1.221762231	2.09948	10.66041	0.00122	0.037101
21	AC008060.1	2.066518	1.846184711	0.412117	4.479763	7.47E-06	1.15E-05	-2.691407753	3.659836	29.05229	1.44E-07	2.15E-05
22	AC008278.1	1.5182806	3.029070028	0.250685	12.0832	1.30E-33	3.81E-33	1.829768289	2.868209	45.03638	9.55E-11	3.39E-08
23	AC008568.1	0.7644705	2.242751378	0.515183	4.35331	1.34E-05	2.04E-05	-1.9593443	2.364716	20.80514	7.41E-06	0.000611
24	AC008938.1	0.9217078	1.744393398	0.218806	7.972346	1.56E-15	3.23E-15	-1.175155499	2.377682	13.95696	0.000224	0.010242
25	AC009060.1	1.4179775	1.941162827	0.296762	6.541147	6.10E-11	1.11E-10	-1.166651164	2.550856	10.3615	0.001427	0.041958
26	AC009123.1	5.8072237	-3.548750299	0.182671	-19.427	4.56E-84	2.73E-83	-1.252005475	1.94167	15.36498	0.00011	0.005756
27	AC009133.3	6.1831796	2.454466191	0.208304	11.78308	4.77E-32	1.37E-31	1.383564927	4.268693	23.65843	1.87E-06	0.000197
28	AC009365.1	0.3613542	1.449159363	0.282719	5.125792	2.96E-07	4.80E-07	2.585686353	1.981697	88.15325	1.52E-18	1.96E-15
29	AC009407.1	1.8560791	1.57794478	0.195658	8.064804	7.34E-16	1.53E-15	1.192915788	2.758238	23.63654	1.87E-06	0.000197
30	AC009988.1	0.6425028	2.513744621	0.373227	6.735156	1.64E-11	3.03E-11	-1.498183269	1.894602	21.45496	5.39E-06	0.000479
31	AC010247.2	8.8079968	2.474013514	0.146009	16.94422	2.12E-64	9.87E-64	1.763081511	4.858685	68.29641	4.53E-15	4.25E-12
32	AC010894.3	21.954856	2.41187281	0.206612	11.67344	1.74E-31	4.94E-31	-1.659901845	5.460169	18.93555	1.86E-05	0.001335
33	AC011474.1	1.8408516	3.640872685	0.393696	9.247926	2.29E-20	5.29E-20	1.504037926	2.869342	19.21117	1.62E-05	0.001186
34	AC012103.1	0.4220554	1.842532898	0.419081	4.396609	1.10E-05	1.68E-05	1.719697357	1.86557	36.47009	4.54E-09	1.06E-06
35	AC012150.1	0.6895919	1.160825073	0.33542	3.460813	0.000539	0.000765	2.442954617	2.31212	66.36182	1.00E-14	7.93E-12
36	AC015522.1	2.1819419	3.832692835	0.359698	10.65532	1.65E-26	4.29E-26	-1.33886656	3.088864	10.94241	0.001055	0.03312
37	AC016885.1	0.3151744	1.408478069	0.446085	3.157419	0.001592	0.002203	1.588672358	1.827275	27.45147	3.03E-07	3.95E-05

NO.	row	Tumor vs Normal						Sensitive vs Resistant				
		baseMean	L2FC	lfcSE	stat	PValue	padj	L2FC	logCPM	F	PValue	FDR
38	AC021192.1	0.4812284	2.167421748	0.357643	6.060297	1.36E-09	2.38E-09	-1.038375257	1.767307	12.15341	0.000563	0.021087
39	AC021218.1	3.7420582	4.336405191	0.281574	15.40058	1.62E-53	6.51E-53	1.069788369	3.583134	12.65866	0.000435	0.017477
40	AC021979.2	0.4399912	1.292699608	0.339782	3.804495	0.000142	0.000207	-1.369649337	1.890734	14.96746	0.000134	0.006641
41	AC022424.1	217.26516	-5.407735742	0.229631	-23.5496	1.27E-122	1.16E-121	-1.470972318	4.188872	13.24894	0.000322	0.013649
42	AC022730.4	5.1262299	-1.327745799	0.289311	-4.58934	4.45E-06	6.91E-06	-2.339531332	3.091427	30.65343	6.76E-08	1.12E-05
43	AC023051.1	5.717323	1.065799626	0.138872	7.674671	1.66E-14	3.34E-14	1.733588081	4.133783	58.91801	2.30E-13	1.39E-10
44	AC023796.1	0.4602121	2.028464184	0.438235	4.628708	3.68E-06	5.74E-06	3.463858376	2.720034	113.538	1.06E-22	3.25E-19
45	AC025569.1	84.898829	-1.654677597	0.160056	-10.3381	4.74E-25	1.21E-24	-1.006851463	5.919034	12.68666	0.000429	0.017333
46	AC034228.1	1.7162292	3.282501872	0.431138	7.613582	2.67E-14	5.33E-14	-2.0581376	2.712518	24.63228	1.17E-06	0.00014
47	AC067960.1	0.8465366	2.786049708	0.417987	6.6654	2.64E-11	4.86E-11	1.725706826	2.326753	31.02712	5.63E-08	9.53E-06
48	AC068489.1	0.4045494	1.84799243	0.448598	4.119481	3.80E-05	5.67E-05	-1.253357272	1.681045	16.22452	7.12E-05	0.00399
49	AC068594.1	0.6018603	2.133756379	0.327716	6.510999	7.47E-11	1.36E-10	1.662496677	2.08904	34.04947	1.38E-08	2.64E-06
50	AC069061.2	1.0670399	2.969072211	0.488982	6.071947	1.26E-09	2.21E-09	-3.285202549	3.229985	40.93063	6.13E-10	1.71E-07
51	AC069285.2	0.7634874	2.792302262	0.575109	4.855261	1.20E-06	1.91E-06	1.782127034	2.774821	23.65442	1.87E-06	0.000197
52	AC080129.1	0.2812047	1.367020939	0.393723	3.472033	0.000517	0.000734	-1.312388314	1.687971	17.71027	3.40E-05	0.002195
53	AC083967.1	21.505884	7.427718957	0.292181	25.42163	1.45E-142	1.65E-141	-1.934803068	6.441636	19.47451	1.43E-05	0.001069
54	AC087379.2	0.4837668	1.686710348	0.383266	4.40089	1.08E-05	1.65E-05	-1.498965693	2.09411	16.41813	6.46E-05	0.003681
55	AC087482.1	5.9565637	-3.244342184	0.135673	-23.9129	2.25E-126	2.14E-125	-1.411646468	2.607391	14.41763	0.000177	0.008355
56	AC087636.1	1.5622195	3.578548386	0.384944	9.296295	1.45E-20	3.37E-20	1.138270882	2.365051	13.26373	0.000318	0.013584
57	AC090044.1	4.860338	-1.770155082	0.21541	-8.21761	2.08E-16	4.40E-16	-1.205282562	2.47683	12.41013	0.000493	0.019108
58	AC090340.1	2.1633745	3.054321013	0.249907	12.22182	2.38E-34	7.09E-34	1.864569313	3.178135	44.25361	1.36E-10	4.24E-08
59	AC090826.2	0.9838	-1.067206857	0.240446	-4.43846	9.06E-06	1.39E-05	1.251380109	1.853728	21.10684	6.39E-06	0.000549
60	AC092155.1	69.242384	-6.495940365	0.153136	-42.4194	0	0	-1.44645186	2.650081	16.45554	6.35E-05	0.003634
61	AC092484.1	17.662577	6.932799332	0.299225	23.16916	9.32E-119	8.17E-118	1.170775783	5.231375	13.70015	0.000256	0.011459
62	AC092747.1	0.4773423	1.844067559	0.295144	6.248022	4.16E-10	7.38E-10	-1.08557252	1.917372	11.90523	0.000639	0.023084
63	AC092834.1	0.4956363	1.263234816	0.382277	3.304502	0.000951	0.001334	1.213012344	2.097475	11.36953	0.000844	0.028133
64	AC092916.1	2.2227436	4.272528205	0.69637	6.135432	8.49E-10	1.50E-09	-2.499619544	3.718867	23.14823	2.40E-06	0.000237
65	AC092954.1	0.8859075	1.958492063	0.349808	5.598769	2.16E-08	3.64E-08	-1.333359715	1.99697	16.53963	6.08E-05	0.003503
66	AC092957.1	2.1688121	4.325274795	0.6293	6.873151	6.28E-12	1.18E-11	-1.51005144	2.977077	11.36165	0.000849	0.028133
67	AC093821.1	4.1473113	4.670892612	0.380524	12.2749	1.24E-34	3.70E-34	2.087376538	4.328059	29.91606	9.63E-08	1.48E-05
68	AC095050.1	0.699074	1.638036651	0.330438	4.957164	7.15E-07	1.14E-06	-1.003216635	1.851614	10.4591	0.001356	0.040383
69	AC097375.1	0.8511835	2.807203711	0.395569	7.096625	1.28E-12	2.45E-12	-1.270510649	2.154079	13.09819	0.000346	0.014514
70	AC097532.1	0.2500223	1.249027077	0.430738	2.899737	0.003735	0.005063	-1.17849531	1.665033	13.27665	0.000316	0.01358
71	AC099509.1	0.6064364	1.40270384	0.361445	3.880818	0.000104	0.000153	-1.292449697	2.095225	11.38712	0.000836	0.02807
72	AC099542.1	1.9822347	-1.720592063	0.163728	-10.5088	7.87E-26	2.03E-25	-1.019026677	1.901772	14.41148	0.000178	0.008355
73	AC100872.2	15.501318	5.449410107	0.263385	20.68989	4.27E-95	2.87E-94	-1.239903486	5.103473	13.4916	0.000284	0.012576
74	AC105383.1	6.2122331	-1.242477937	0.153972	-8.06953	7.06E-16	1.48E-15	-1.039151486	3.202657	12.34227	0.00051	0.019633

NO.	row	Tumor vs Normal						Sensitive vs Resistant				
		baseMean	L2FC	lfcSE	stat	PValue	padj	L2FC	logCPM	F	PValue	FDR
75	AC105460.1	1.1433753	3.384119908	0.366659	9.229608	2.72E-20	6.27E-20	1.766758642	3.016258	28.16538	2.18E-07	2.99E-05
76	AC108136.1	0.748076	2.676435729	0.380287	7.037941	1.95E-12	3.71E-12	-1.228149211	2.146209	12.04233	0.000596	0.021942
77	AC108868.1	0.4692068	2.094431968	0.442224	4.73613	2.18E-06	3.42E-06	-2.944774271	2.872975	37.81031	2.50E-09	6.29E-07
78	AC108868.2	0.5680523	2.306922443	0.56155	4.108131	3.99E-05	5.95E-05	-2.464533144	2.217378	32.67911	2.61E-08	4.81E-06
79	AC109492.1	0.8789663	3.022007133	0.515757	5.859359	4.65E-09	7.99E-09	-1.598968911	2.350535	15.36029	0.00011	0.005756
80	AC113194.1	1.1081938	2.25794887	0.384882	5.8666	4.45E-09	7.66E-09	-1.894181419	2.256582	24.8806	1.03E-06	0.000126
81	AC114284.1	6.425929	-2.637752536	0.190226	-13.8664	1.01E-43	3.51E-43	-1.014701239	2.739215	9.935136	0.001784	0.049975
82	AC121154.1	0.6515727	2.099741589	0.393859	5.331195	9.76E-08	1.61E-07	-1.13710792	1.998009	10.32775	0.001452	0.042398
83	AC124067.3	0.5444624	1.469996439	0.401204	3.663959	0.000248	0.000358	-2.071091735	2.387684	23.57025	1.94E-06	0.000202
84	AC125616.1	2.3312716	2.697201137	0.300341	8.980474	2.70E-19	6.11E-19	-1.124731649	2.75483	10.60731	0.001255	0.037927
85	AC129507.1	209.13811	-1.363494501	0.149434	-9.1244	7.21E-20	1.65E-19	-1.092303427	7.433568	15.08049	0.000127	0.006391
86	AC130456.4	1.2998104	3.596623974	0.300696	11.961	5.69E-33	1.65E-32	1.055612421	2.635421	12.78958	0.000406	0.01666
87	AC131094.1	2.4539264	4.478820768	0.432893	10.34625	4.35E-25	1.11E-24	-2.741497091	4.053894	30.55386	7.14E-08	1.15E-05
88	AC134312.1	37.027883	-1.915255432	0.177794	-10.7723	4.65E-27	1.22E-26	1.056234598	4.588292	18.53684	2.26E-05	0.001564
89	AC147651.3	0.7043183	2.123085006	0.384201	5.525976	3.28E-08	5.49E-08	1.882577855	2.264237	37.32149	3.08E-09	7.38E-07
90	AC246787.2	2.0493995	3.923480874	0.341358	11.49374	1.42E-30	3.96E-30	-1.105818922	2.638233	10.07686	0.001656	0.047161
91	AF106564.1	4.8010938	-1.552232029	0.256757	-6.04554	1.49E-09	2.60E-09	1.0311124	2.510092	10.14497	0.001598	0.04601
92	AGBL1-AS1	0.6624141	1.768267062	0.316182	5.592566	2.24E-08	3.77E-08	-1.823920196	2.441751	21.05379	6.56E-06	0.000556
93	AL033504.1	1.0930701	1.792557557	0.341478	5.249403	1.53E-07	2.50E-07	-3.117096356	3.488918	40.72907	6.72E-10	1.82E-07
94	AL033527.2	5.6879938	5.156377049	0.210721	24.47016	3.07E-132	3.13E-131	-1.42512185	4.843253	18.64721	2.15E-05	0.001495
95	AL079303.1	6.6458283	3.279162557	0.302156	10.85253	1.94E-27	5.14E-27	-1.714549944	4.081209	19.54248	1.38E-05	0.00104
96	AL109610.1	0.5149029	2.266309414	0.553949	4.091188	4.29E-05	6.40E-05	1.531556661	1.904255	24.25618	1.39E-06	0.000163
97	AL117372.1	11.971737	5.721593897	0.34917	16.38628	2.40E-60	1.06E-59	-1.44076904	5.222711	10.90505	0.001077	0.033541
98	AL122034.1	1.4691196	3.252080593	0.290974	11.17654	5.31E-29	1.45E-28	1.827853566	2.813245	39.10664	1.37E-09	3.62E-07
99	AL133232.1	0.2832602	1.35026327	0.381065	3.543394	0.000395	0.000565	-2.084359013	2.36428	23.93018	1.63E-06	0.000181
100	AL133370.1	9.3521363	3.81978097	0.250028	15.27743	1.08E-52	4.28E-52	-2.389723497	4.919609	35.213	8.26E-09	1.70E-06
101	AL136962.1	26.957276	4.673829729	0.209	22.36286	9.05E-111	7.20E-110	1.021573756	6.087471	12.45618	0.000483	0.018998
102	AL138885.2	2.193716	4.3689348	0.297678	14.67672	9.09E-49	3.41E-48	-1.071867356	2.778552	11.58993	0.000753	0.026027
103	AL138930.1	0.6440267	2.149235161	0.308677	6.962733	3.34E-12	6.31E-12	-1.025550251	2.031355	9.957462	0.001763	0.049526
104	AL139002.1	1.1826239	2.826552693	0.406991	6.945001	3.78E-12	7.15E-12	-1.728247389	2.992524	15.81611	8.77E-05	0.004733
105	AL160262.1	0.6428983	2.533241034	0.419051	6.045181	1.49E-09	2.61E-09	-1.383571467	2.062665	14.70497	0.000153	0.007415
106	AL162713.1	2.0766692	-2.506883033	0.169992	-14.747	3.21E-49	1.21E-48	1.108956197	1.743351	18.74302	2.04E-05	0.001438
107	AL353681.1	0.7905656	1.063296806	0.280986	3.784164	0.000154	0.000224	1.37844773	2.081502	22.5288	3.20E-06	0.000308
108	AL354714.1	7.2117586	-3.194907995	0.149936	-21.3085	9.47E-101	6.80E-100	-1.211981335	2.475472	15.84958	8.60E-05	0.004711
109	AL354864.1	0.8100215	2.405558749	0.244499	9.838741	7.67E-23	1.87E-22	-4.047094237	4.451374	53.2982	2.68E-12	1.38E-09
110	AL356804.1	0.8433338	1.81567645	0.369176	4.918181	8.74E-07	1.39E-06	-2.19543211	2.486799	27.98551	2.36E-07	3.20E-05
111	AL359378.1	0.6388923	2.474494188	0.365048	6.778541	1.21E-11	2.26E-11	2.078910371	2.998356	35.60952	6.83E-09	1.53E-06

NO.	row	Tumor vs Normal						Sensitive vs Resistant				
		baseMean	L2FC	lfcSE	stat	PValue	padj	L2FC	logCPM	F	PValue	FDR
112	AL360169.1	1.5502966	3.870507203	0.330441	11.71317	1.09E-31	3.10E-31	1.263491057	2.808159	16.55402	6.05E-05	0.003503
113	AL390786.1	1.2294135	3.51100841	0.417678	8.406023	4.24E-17	9.16E-17	-1.381188275	2.554391	13.21719	0.000326	0.013778
114	AL391361.2	0.6052014	1.450632859	0.302091	4.801973	1.57E-06	2.48E-06	1.640311615	2.059579	33.06932	2.18E-08	4.08E-06
115	AL391704.1	1.0584706	3.238970435	0.391113	8.281414	1.22E-16	2.60E-16	-1.761447245	2.823278	18.06238	2.86E-05	0.001927
116	AL445465.1	1.8840498	2.528797185	0.268311	9.424889	4.31E-21	1.01E-20	-1.888698122	3.299684	24.0281	1.56E-06	0.000175
117	AL449403.1	3.8294016	-3.438828516	0.181928	-18.9021	1.10E-79	6.24E-79	-1.962586383	2.383159	23.72445	1.80E-06	0.000195
118	AL451048.1	0.6393611	2.308171471	0.417909	5.523148	3.33E-08	5.58E-08	1.368847404	1.850607	22.25816	3.65E-06	0.000342
119	AL499627.1	0.4768485	1.570773176	0.40362	3.891714	9.95E-05	0.000146	-1.237011067	1.7074	15.06159	0.000128	0.006394
120	AL513324.1	0.774458	2.566538709	0.322667	7.954138	1.80E-15	3.73E-15	1.309966307	2.23831	19.93601	1.13E-05	0.000871
121	AL513542.1	0.3964636	1.445933409	0.389373	3.713494	0.000204	0.000296	1.176150095	1.631138	20.12159	1.03E-05	0.000801
122	AL583785.1	20.673176	1.430157834	0.257659	5.550584	2.85E-08	4.78E-08	-1.345644757	6.031736	10.60318	0.001261	0.037992
123	AL591686.1	0.9202696	1.44278644	0.366392	3.937824	8.22E-05	0.000121	1.240691914	2.077997	15.8397	8.64E-05	0.004711
124	AL606519.1	1.3412718	3.649296368	0.35183	10.37232	3.31E-25	8.45E-25	-1.141089739	2.331646	11.57059	0.00076	0.026033
125	AL683887.1	0.6930242	2.6506022	0.37792	7.013663	2.32E-12	4.41E-12	-1.072789457	1.937887	11.28683	0.000881	0.028818
126	AL731537.1	3.2129074	-1.904957091	0.187989	-10.1334	3.93E-24	9.82E-24	1.499906792	2.111834	30.05425	8.88E-08	1.41E-05
127	AP003774.2	6.0337497	1.513167667	0.122045	12.39841	2.67E-35	8.08E-35	1.23644129	3.985231	42.60455	2.83E-10	8.32E-08
128	AP007216.2	1.483861	2.269154975	0.243024	9.337163	9.90E-21	2.30E-20	1.852616421	2.726755	48.55448	2.02E-11	8.32E-09
129	BX546450.1	0.4379515	1.184138895	0.414338	2.857903	0.004265	0.00576	-1.51356502	1.814687	18.88137	1.90E-05	0.001352
130	C10orf71-AS1	0.6338645	1.970674694	0.502955	3.918192	8.92E-05	0.000131	-1.802126138	1.790394	24.9995	9.74E-07	0.000121
131	C20orf78	0.3415862	1.158342496	0.398645	2.9057	0.003664	0.004971	-1.380568854	1.587453	22.11852	3.90E-06	0.000359
132	CELF2-AS1	59.229639	-4.734319674	0.127679	-37.0799	5.92E-301	2.79E-299	1.261198664	3.624464	24.27676	1.38E-06	0.000163
133	CISTR	1.338049	-1.105833686	0.279203	-3.96068	7.47E-05	0.00011	2.486511127	2.052523	66.58521	9.12E-15	7.83E-12
134	ERICH3-AS1	0.8063208	2.675805815	0.486043	5.505289	3.69E-08	6.17E-08	-1.727271867	2.094258	20.16435	1.01E-05	0.00079
135	ERVK-28	1.5230476	2.859840934	0.341493	8.374526	5.54E-17	1.19E-16	-1.515034185	2.397769	18.34428	2.48E-05	0.001687
136	FAM167A-AS1	1.487271	-1.630766079	0.376339	-4.33324	1.47E-05	2.23E-05	-3.668287996	3.731895	44.13619	1.47E-10	4.46E-08
137	FARSA-AS1	4.7853693	2.796600016	0.153486	18.22058	3.54E-74	1.88E-73	3.416829085	5.100077	190.3937	7.90E-34	8.14E-30
138	FP671120.6	254.03021	8.185049139	0.210502	38.88342	0	0	1.102766076	8.915997	16.28386	6.95E-05	0.003938
139	FRMD6-AS2	19.898573	-5.679739172	0.192638	-29.484	4.62E-191	8.41E-190	1.159471085	1.870044	15.21772	0.000118	0.006087
140	GACAT3	5.5013372	-1.868285515	0.176746	-10.5705	4.08E-26	1.06E-25	-1.294571895	2.546019	16.75439	5.47E-05	0.003275
141	GATA3-AS1	1.4825983	2.717178509	0.285324	9.523146	1.68E-21	3.97E-21	1.461409837	2.784744	23.49174	2.01E-06	0.000207
142	GPR50-AS1	0.8478949	2.607015383	0.489802	5.322587	1.02E-07	1.69E-07	-1.703672202	2.775041	14.81092	0.000145	0.007171
143	HNF1A-AS1	9.0023562	4.508162293	0.282272	15.97097	2.04E-57	8.59E-57	-1.724940723	6.374451	11.85335	0.000659	0.02326
144	HOXA-AS3	25.841176	2.283967805	0.222273	10.27552	9.09E-25	2.30E-24	1.082151468	5.541914	12.08753	0.000584	0.021722
145	IGFL2-AS1	74.043854	4.572749353	0.223047	20.50125	2.10E-93	1.39E-92	1.40631956	7.422344	16.85521	5.23E-05	0.00317
146	LINC00112	0.4779102	2.127667338	0.335773	6.336627	2.35E-10	4.19E-10	1.146703877	1.955935	15.18314	0.00012	0.006152
147	LINC00314	2.2546199	3.666413803	0.324175	11.31	1.17E-29	3.23E-29	-1.092090473	2.788136	10.50783	0.001322	0.039486
148	LINC00348	1.3740288	3.723530591	0.57696	6.453705	1.09E-10	1.97E-10	-1.919739878	2.385511	21.5057	5.27E-06	0.000472

NO.	row	Tumor vs Normal						Sensitive vs Resistant				
		baseMean	L2FC	lfcSE	stat	PValue	padj	L2FC	logCPM	F	PValue	FDR
149	LINC00390	1.2794845	1.913829428	0.358993	5.3311	9.76E-08	1.61E-07	1.511339246	2.502165	21.70767	4.77E-06	0.000431
150	LINC00393	5.1328755	5.123472644	0.391902	13.07334	4.68E-39	1.51E-38	-1.349402594	3.871736	10.79328	0.001141	0.035108
151	LINC00477	0.4955193	1.835675406	0.405494	4.527012	5.98E-06	9.26E-06	-1.587298697	1.844211	21.1593	6.23E-06	0.000539
152	LINC00639	40.178451	1.432169442	0.160196	8.940129	3.89E-19	8.78E-19	-1.013444552	6.227199	12.51458	0.000469	0.018638
153	LINC00645	9.1562372	5.802375218	0.332408	17.45556	3.12E-68	1.54E-67	-1.819949689	5.433185	16.19528	7.27E-05	0.00405
154	LINC00836	3.8659102	4.884933686	0.361746	13.50376	1.49E-41	4.99E-41	-3.549559671	5.061299	44.61999	1.19E-10	3.98E-08
155	LINC01151	6.8069363	5.156951175	0.301281	17.11677	1.11E-65	5.27E-65	-1.171422648	4.026148	11.13828	0.000953	0.030895
156	LINC01159	1.5224049	2.887914528	0.475555	6.072719	1.26E-09	2.20E-09	3.972704456	3.768842	117.5599	2.74E-23	1.41E-19
157	LINC01160	32.221045	-1.594721783	0.118604	-13.4457	3.26E-41	1.09E-40	1.186491128	4.913881	34.75171	1.00E-08	2.02E-06
158	LINC01229	26.274768	-4.268295557	0.122014	-34.982	4.22E-268	1.53E-266	-1.934538035	3.585547	29.89461	9.64E-08	1.48E-05
159	LINC01230	2.3302402	1.776742278	0.311717	5.699856	1.20E-08	2.03E-08	-1.590099588	2.903203	17.66776	3.47E-05	0.002211
160	LINC01266	9.1356513	-1.005323139	0.217024	-4.63232	3.62E-06	5.64E-06	-1.512062323	3.414286	17.86762	3.15E-05	0.002068
161	LINC01419	0.9004813	2.907703521	0.445758	6.523049	6.89E-11	1.25E-10	-3.768292446	3.307347	50.47239	9.01E-12	3.94E-09
162	LINC01541	4.6360533	2.074108171	0.587489	3.530462	0.000415	0.000592	-4.977498611	6.167386	50.84709	7.74E-12	3.63E-09
163	LINC01553	1.3030028	3.144716281	0.243672	12.90553	4.19E-38	1.33E-37	-1.833382172	3.161401	24.22019	1.42E-06	0.000163
164	LINC01593	0.5483475	1.966616708	0.456028	4.31249	1.61E-05	2.45E-05	-1.076201199	1.777035	10.56947	0.00128	0.03834
165	LINC01632	0.7483542	2.720114042	0.465363	5.845145	5.06E-09	8.70E-09	1.097454553	2.132892	11.11038	0.000965	0.030994
166	LINC01639	4.6112955	4.736889948	0.460381	10.28907	7.89E-25	2.00E-24	1.23074612	3.523083	10.83397	0.001117	0.034577
167	LINC01673	0.5386358	2.383053321	0.420701	5.664477	1.47E-08	2.49E-08	-1.557002438	1.828345	23.38825	2.11E-06	0.000211
168	LINC01693	2.0577995	3.551381511	0.314357	11.2973	1.35E-29	3.72E-29	1.27029976	2.889343	16.94515	4.97E-05	0.003052
169	LINC01722	2.2030384	2.721352363	0.281486	9.667793	4.13E-22	9.90E-22	1.185582459	2.700582	17.42199	3.92E-05	0.002448
170	LINC01754	1.5525494	1.516788223	0.247249	6.13467	8.53E-10	1.50E-09	1.159532463	2.521451	15.79434	8.84E-05	0.004744
171	LINC01767	5.233724	2.54180946	0.17949	14.16126	1.59E-45	5.70E-45	-2.091647589	5.160678	27.5968	2.86E-07	3.83E-05
172	LINC01777	1.2312413	3.301189001	0.387079	8.528458	1.48E-17	3.24E-17	-1.299843716	2.562872	12.01073	0.000606	0.022222
173	LINC01785	10.100094	-2.349189886	0.218489	-10.752	5.80E-27	1.52E-26	1.220126589	3.208571	12.2376	0.00054	0.020457
174	LINC01793	2.5334007	2.928742932	0.377218	7.764066	8.22E-15	1.67E-14	1.532227755	4.920304	12.45872	0.000483	0.018998
175	LINC01887	1.5510649	3.68474075	0.2532	14.5527	5.61E-48	2.09E-47	-1.116320161	2.730535	13.52227	0.000279	0.012393
176	LINC01897	0.7588338	2.734794625	0.405051	6.751724	1.46E-11	2.71E-11	1.05374476	1.917064	12.3613	0.000506	0.019515
177	LINC01926	0.3655108	1.433320244	0.369411	3.880012	0.000104	0.000153	-1.154006387	1.729746	12.74978	0.000414	0.016869
178	LINC02006	14.71785	1.27745397	0.15758	8.106686	5.20E-16	1.09E-15	-1.076990752	4.701559	18.69104	2.09E-05	0.001467
179	LINC02020	0.7046257	2.562513261	0.50684	5.055867	4.28E-07	6.91E-07	-1.971733525	2.2939	22.32922	3.53E-06	0.000337
180	LINC02038	2.2463443	4.298854017	0.429334	10.01285	1.34E-23	3.31E-23	-1.644965073	3.144195	15.0338	0.00013	0.006473
181	LINC02109	2.5282725	3.957342471	0.428756	9.229834	2.71E-20	6.26E-20	1.213875845	3.193803	11.01758	0.001015	0.032187
182	LINC02119	1.3064045	-1.516404673	0.248086	-6.11242	9.81E-10	1.73E-09	1.505076046	2.038524	21.92998	4.28E-06	0.00039
183	LINC02154	6.418962	4.802921919	0.245231	19.58529	2.06E-85	1.25E-84	-1.234281844	4.716773	12.54529	0.000461	0.018425
184	LINC02160	1.3448133	3.459246982	0.364688	9.485492	2.41E-21	5.68E-21	-2.639905011	3.240823	34.70557	1.04E-08	2.05E-06
185	LINC02167	39.149917	8.423611482	0.330451	25.49126	2.46E-143	2.81E-142	-1.954434133	7.397899	16.24134	7.10E-05	0.00399

NO.	row	Tumor vs Normal						Sensitive vs Resistant				
		baseMean	L2FC	lfcSE	stat	PValue	padj	L2FC	logCPM	F	PValue	FDR
186	LINC02266	2.2847687	2.670814266	0.26654	10.02033	1.24E-23	3.07E-23	-1.845244117	3.294447	24.23353	1.41E-06	0.000163
187	LINC02331	11.370689	5.7359924	0.290962	19.7139	1.64E-86	1.01E-85	1.177915775	4.643964	15.30091	0.000114	0.00589
188	LINC02343	1.0948385	3.245285132	0.350284	9.264722	1.96E-20	4.53E-20	2.805856968	2.943858	82.56641	1.41E-17	1.62E-14
189	LINC02346	6.3895939	5.673423519	0.288091	19.69318	2.47E-86	1.51E-85	2.380017917	4.603083	61.25771	8.93E-14	5.75E-11
190	LINC02365	3.7289013	4.526838908	0.307509	14.72101	4.73E-49	1.78E-48	1.439677489	3.829534	19.56276	1.37E-05	0.00104
191	LINC02388	23.052045	-3.892065055	0.235418	-16.5326	2.14E-61	9.57E-61	-1.384855371	2.809998	16.76114	5.45E-05	0.003275
192	LINC02400	0.2793402	1.132434656	0.469153	2.413784	0.015788	0.020582	3.02221642	2.072537	104.2505	3.19E-21	4.70E-18
193	LINC02404	0.7376416	1.87768375	0.350942	5.350406	8.78E-08	1.45E-07	-1.919492302	2.224255	25.81067	6.61E-07	8.30E-05
194	LINC02434	1.3942391	3.621452521	0.631372	5.735848	9.70E-09	1.65E-08	1.49720105	3.307465	14.51533	0.000169	0.008068
195	LINC02435	3.6951108	2.307745574	0.213419	10.81322	2.98E-27	7.87E-27	1.040235918	3.293712	15.15976	0.000122	0.006176
196	LINC02455	0.3862404	1.46629257	0.312196	4.696711	2.64E-06	4.14E-06	-1.572430223	1.990263	19.53846	1.38E-05	0.00104
197	LINC02489	1.2123609	-2.211908238	0.23004	-9.6153	6.89E-22	1.64E-21	-1.707348728	1.794657	22.21629	3.72E-06	0.000346
198	LINC02582	1.1698551	2.342390162	0.342357	6.841953	7.81E-12	1.46E-11	3.632333278	3.638142	109.4955	5.10E-22	9.68E-19
199	LINC02623	0.3040792	1.194864247	0.407327	2.933424	0.003352	0.004558	2.241426124	1.919123	57.53498	4.13E-13	2.36E-10
200	LINC02660	0.7206736	1.40151389	0.341109	4.108693	3.98E-05	5.94E-05	1.292853041	1.939662	20.36265	9.19E-06	0.000734
201	LINC02753	0.5354259	1.521195519	0.436951	3.481386	0.000499	0.000709	1.670359651	1.914683	32.23444	3.21E-08	5.70E-06
202	LINGO1-AS1	3.8763031	-1.216092737	0.162301	-7.49283	6.74E-14	1.33E-13	-1.551248533	3.537804	16.71804	5.59E-05	0.003328
203	MACC1-AS1	2.2531878	-1.961323092	0.241638	-8.1168	4.79E-16	1.01E-15	-4.436964101	5.573495	45.34977	8.62E-11	3.17E-08
204	MAPT-AS1	2.4868358	-3.414411295	0.179183	-19.0554	5.93E-81	3.41E-80	1.396651246	1.618947	29.20587	1.32E-07	2.00E-05
205	MIR137HG	7.7686999	-3.363704573	0.241617	-13.9216	4.68E-44	1.63E-43	-1.356180566	2.170404	15.3024	0.000113	0.00589
206	MKX-AS1	6.5038759	-2.660284337	0.175821	-15.1306	1.02E-51	3.97E-51	2.117956394	2.871566	45.50721	7.81E-11	3.10E-08
207	MNX1-AS1	9.7248262	5.483039285	0.345771	15.85742	1.25E-56	5.23E-56	-1.708212075	5.063799	14.71159	0.000153	0.007415
208	NDP-AS1	1.1897343	1.332187814	0.280192	4.75456	1.99E-06	3.13E-06	-1.914451653	2.732771	22.64565	3.03E-06	0.000295
209	NGF-AS1	3.5832859	-2.446169657	0.166861	-14.6599	1.16E-48	4.36E-48	-3.062214318	4.033001	36.04342	5.65E-09	1.29E-06
210	OVAAL	31.407139	6.8502634	0.23988	28.55704	2.30E-179	3.72E-178	1.297310236	6.75944	17.78622	3.29E-05	0.002147
211	PCAT14	242.23265	-3.052382289	0.166536	-18.3287	4.89E-75	2.62E-74	1.415910787	6.831577	23.46438	2.06E-06	0.000208
212	PINCR	1.1481895	3.400469112	0.533253	6.376845	1.81E-10	3.24E-10	-1.601523889	3.380174	11.44815	0.000812	0.027354
213	PKIA-AS1	12.330221	-1.20596644	0.202967	-5.94167	2.82E-09	4.89E-09	-1.172176188	3.680883	14.55231	0.000165	0.007971
214	PLCH1-AS1	0.281337	1.378708674	0.406697	3.390018	0.000699	0.000987	-1.106614413	1.56946	16.54357	6.07E-05	0.003503
215	PROX1-AS1	12.642316	-2.432773496	0.167528	-14.5216	8.84E-48	3.28E-47	1.622737057	3.556492	28.89639	1.54E-07	2.27E-05
216	RORB-AS1	3.4583728	2.004943932	0.273187	7.339093	2.15E-13	4.20E-13	2.360603869	4.283446	44.53946	1.23E-10	3.98E-08
217	SIRLNT	0.3215783	1.3087273	0.509166	2.570337	0.01016	0.013437	-1.751413188	2.177017	16.61653	5.86E-05	0.003471
218	TDRG1	7.606614	1.079154571	0.227152	4.750802	2.03E-06	3.18E-06	1.559150615	4.596575	19.12827	1.70E-05	0.001222
219	TMEM132D-A	2.6425007	4.136571256	0.468999	8.819997	1.14E-18	2.56E-18	3.258336968	4.809328	66.0823	1.21E-14	8.89E-12
220	U95743.1	3.3561186	4.906968835	0.495362	9.90582	3.93E-23	9.61E-23	1.13857793	3.19652	10.03412	0.001696	0.048037
